# IL-12 Directs Further Maturation of *Ex Vivo* Differentiated NK Cells with Improved Therapeutic Potential

**DOI:** 10.1371/journal.pone.0087131

**Published:** 2014-01-31

**Authors:** Dorit Lehmann, Jan Spanholtz, Caterina Sturtzel, Marleen Tordoir, Bernhard Schlechta, Dirk Groenewegen, Erhard Hofer

**Affiliations:** 1 Department of Vascular Biology and Thrombosis Research, Center for Physiology and Pharmacology, Medical University of Vienna, Vienna, Austria; 2 Glycostem Therapeutics, s-Hertogenbosch, Nijmegen, The Netherlands; 3 Department of Obstetrics and Gynecology, Medical University of Vienna, Vienna, Austria; University of Minnesota, United States of America

## Abstract

The possibility to modulate *ex vivo* human NK cell differentiation towards specific phenotypes will contribute to a better understanding of NK cell differentiation and facilitate tailored production of NK cells for immunotherapy. In this study, we show that addition of a specific low dose of IL-12 to an *ex vivo* NK cell differentiation system from cord blood CD34^+^ stem cells will result in significantly increased proportions of cells with expression of CD62L as well as KIRs and CD16 which are preferentially expressed on mature CD56^dim^ peripheral blood NK cells. In addition, the cells displayed decreased expression of receptors such as CCR6 and CXCR3, which are typically expressed to a lower extent by CD56^dim^ than CD56^bright^ peripheral blood NK cells. The increased number of CD62L and KIR positive cells prevailed in a population of CD33^+^NKG2A^+^ NK cells, supporting that maturation occurs via this subtype. Among a series of transcription factors tested we found Gata3 and TOX to be significantly downregulated, whereas ID3 was upregulated in the IL-12-modulated *ex vivo* NK cells, implicating these factors in the observed changes. Importantly, the cells differentiated in the presence of IL-12 showed enhanced cytokine production and cytolytic activity against MHC class I negative and positive targets. Moreover, in line with the enhanced CD16 expression, these cells exhibited improved antibody-dependent cellular cytotoxicity for B-cell leukemia target cells in the presence of the clinically applied antibody rituximab. Altogether, these data provide evidence that IL-12 directs human *ex vivo* NK cell differentiation towards more mature NK cells with improved properties for potential cancer therapies.

## Introduction

Natural killer (NK) cells are innate lymphocytes that exhibit cytotoxic and immunoregulatory functions upon activation. In humans these functions are correlated with two distinct NK cell phenotypes, namely the preferentially cytokine producing CD56^bright^ NK cells that are most prominently found in secondary lymphoid tissues and the blood resident CD56^dim^ NK cells preferentially exerting killing of virus-infected and transformed cells [1]–[3]. Both NK cell subtypes express a typical range of activating and inhibiting receptors balancing their activity. CD56^dim^ NK cells exhibit to a significant extent surface expression of multiple killer cell immunoglobulin-like receptors (KIRs) and are largely positive for CD16 (FcRγIII), the receptor mediating antibody-dependent cellular cytotoxicity (ADCC). In contrast CD56^bright^ NK cells mostly lack the expression of these receptors but reveal to a high percentage expression of the inhibitory receptor CD94/NKG2A. Several indications led to the concept of a stepwise maturation of CD56^bright^ NK cells towards a CD56^dim^ phenotype and function of NK cells [2], [4], [5]. Furthermore, related to the tissue homing sites of these two NK cell subsets a differing expression of chemokine receptors and adhesion molecules was identified. Whereas CCR7 is exclusively expressed on CD56^bright^ NK cells and CD62L, CCR6 and CXCR3 are much more prominent on CD56^bright^ than CD56^dim^ NK cells, other receptors such as CXCR4 exhibit similar expression on both CD56^bright^ and CD56^dim^ adult peripheral blood NK cells [2], [6]–[8].

Several cytokines exert significant biological effects on NK cells. Among those, interleukin 12 (IL-12), which is mainly produced by activated monocytes, macrophages, dendritic cells and B-cells, was shown to induce production of cytokines such as IFN-γ and to enhance cytotoxicity of peripheral blood NK cells [9], [10]. In addition, it has been demonstrated that IL-12 also influences the receptor expression of peripheral blood NK cells. Some earlier studies revealed an induction of the CD56^bright^ NK cell phenotype by IL-12, including an upregulation of CD94 and CD62L and a downmodulation of CD16 [11]. More recently, an upregulation of NKG2A^+^ on NKG2C^+^ NK cells was shown [12].

Since human NK cell differentiation is difficult to study *in vivo*, reliable *ex vivo* differentiation systems are important to gain insights into human NK cell development. Furthermore, NK cells amplified *ex vivo* have been described as promising effectors for adoptive immunotherapy of cancer [13], [14]. Our recently established and characterized *ex vivo* human NK cell differentiation system constitutes a reliable tool to study human NK cell differentiation and provides a promising NK cell product for clinical therapies due to the purity, expansion rates and functional state of the generated NK cells [15]–[17]. Within this system starting from approximately 1×10^5^ CD34^+^ cells obtained from one umbilical cord blood unit more than 10^9^ NK cells can be generated for therapeutic application [15], [16].

In the present study, we show for the first time a detailed impact of IL-12 during *ex vivo* differentiation of progenitors to NK cells in a human system. We found, that preferentially low doses of IL-12 induce the generation of increased proportions of cells with expression of CD62L, CD16 and KIRs and a specific chemokine receptor repertoire without significantly affecting the amplification of the cells during differentiation. Furthermore, we show that mRNA levels for the transcription factors Gata3, TOX and ID3 are significantly changed upon IL-12 addition supporting a potential role of these factors in the IL-12 effects. Moreover, the generated NK cells display improved functions in regard of IFN-γ production as well as cytotoxicity and ADCC-mediated lysis. Altogether, these analyses highlight the impact of IL-12 on human NK cell differentiation and the potential of IL-12 to generate an NK cell product with improved properties for potential therapeutic applications in malignancies.

## Materials and Methods

### Ethics statement

Human umbilical cord blood samples were obtained at birth after full-term delivery from the Department of Obstetrics and Gynecology of the University Hospital of Vienna, Austria, or from the cord blood bank of the Radboud University Nijmegen Medical Center, The Netherlands. Procedures of cord blood collection including a written informed consent have been approved for this study by the corresponding ethical committees of the Medical University of Vienna and of the Radboud University, Nijmegen.

### Isolation of cord blood mononuclear cells (CBMC) and enrichment of CD34^+^ stem and progenitor cells

Mononuclear cells were isolated from the umbilical cord blood samples by density gradient centrifugation (LSM 1077 Lymphocyte Separation Medium, PAA Laboratories GmbH, Graz, Austria) and labelled with CliniMACS CD34 reagent (Miltenyi Biotech, Bergisch-Gladbach, Germany). The selection of CD34^+^ cells was performed according to the manufacturer's instructions. After the enrichment procedure, the CD34^+^ cell fraction was collected and cell number and purity were analyzed by flow cytometry. Finally, the obtained CD34^+^ umbilical cord blood cells were used for the NK cell generation process.

### *Ex vivo* expansion and differentiation of CD34^+^ progenitor cells

CD34^+^ umbilical cord blood cells were transferred into culture plates and expanded and differentiated according to culture method III as described previously [17]. In short, CD34^+^ cells were expanded for 10 days in GBGM® supplemented with a high dose of the factors SCF (27 ng/ml, CellGenix, Freiburg, Germany), IL-7 (25 ng/ml, Stemcell Technologies, Grenoble, France), TPO (25 ng/ml, Stemcell Technologies), Flt3L (25 ng/ml, CellGenix) and a low dose of the factors G-CSF (250 pg/ml, Stemcell Technologies), GM-CSF (10 pg/ml, Stemcell Technologies) and IL-6 (50 pg/ml, CellGenix). Differentiation was induced by replacing TPO by IL-15 (20 ng/ml, CellGenix) at day 10, furthermore Flt3L was replaced by IL-2 (1000 U/ml, Chiron, München, Germany) at day 14. All other cytokines where constantly present until the end of the culture period. During the first 14 days of culture low molecular weight heparin (25 mg/ml, Abbott, Wiesbaden, Germany) was included in the growth medium. Cells were grown for at least 28 days as indicated. rh-IL-12 (Immunotools, Friesoythe, Germany) at a concentration of 0.2 ng/ml (if not indicated differently) was added from day 10 on together with IL-15. Every 3 days fresh culture medium including the described cytokines was added.

For functional studies and realtime RT-PCR analysis the *ex vivo* generated NK cells were purified using CD56 microbeads (Miltenyi Biotec) according the manufacturer's instructions and directly used in functional assays or for RNA extraction.

### Cell lines

Cell lines K562, KG1a and THP-1, obtained from the American Type Culture Collection (human promyeloblast cell line KG-1a, ATTC-CCL-246.1, human monocyte cell line THP-1, ATCC-TIB-202, human lymphoblast cell line K562, ATCC-CCL-243), were cultured in Iscove's modified Dulbecco's medium (IMDM; Invitrogen, Carlsbad CA, USA) containing 50 U/ml penicillin, 50 µg/ml streptomycin and 10% fetal calf serum (FCS; Integro, Zaandam, The Netherlands).

The CD20 expressing human acute lymphoblastic leukemia cell lines 721.221 [18] (a gift of Dr. M. Lopez-Botet; UPF, Barcelona, Spain) and Nalm-6 [19] (kindly provided by Dr. R. Panzer-Grümayer; CCRI St. Anna, Vienna, Austria) were cultured in RPMI-1640 (Sigma-Aldrich, Vienna, Austria) containing 50 U/ml penicillin, 50 µg/ml streptomycin (PAA Laboratories GmbH, Graz, Austria) and 10% FCS.

Lymphatic endothelial cells stably transfected with hTERT (LecTERT) [20] were kindly provided by Dr. P. Petzelbauer, Medical University of Vienna, Austria, cultured in DMEM medium (Invitrogen, Fisher Scientific GmbH, Vienna, Austria) containing penicillin, streptomycin, 20% FCS and 100 µg/ml hygromycin (Invitrogen, Fisher Scientific GmbH, Vienna, Austria).

Human umbilical vein endothelial cells (HUVECs) were isolated as described previously [21] and cultured in EGM-2 medium (Bio Whittacker, Lonza, Verviers, Belgium).

### Flow cytometry

Cell numbers and expression of cell-surface markers were determined by flow cytometry as described previously [15]–[17]. For immunophenotypical staining, cells were first incubated with FcR-blocking reagent (Miltenyi Biotec), followed by the antibodies at the appropriate concentration for 30 min at 4°C. After washing, expression was measured using a FACSCalibur and analyzed with CellQuestPro software (both from BD Biosciences, San Jose, CA). To determine purity and phenotype of the cultured cells the following antibodies were used: CD33 clone D3HL60.251 and NKG2A-PE clone Z199.1.10 (both Beckman Coulter; Marseille, France), CD3-FITC clone UCHT1, CD62L-FITC clone LT-TD180 (all Immunotools; Friesoythe, Germany), CD56-APC clone NCAM16.2, CD16-PE clone 3G8 (all BD Biosciences), KIR-FITC clone 180704 (binding to KIR2DL2, KIR2DL3, KIR2DS2 and KIR2DS4), CXCR3-PE clone 49801, CXCR5-PE clone 51505.111, CCR7-FITC clone 150503 (all R&D Systems; Abingdon, UK), CXCR4-PE clone 12G5, CCR6-Alexa488 clone TG7/CCR6 (all Biolegend; ITK Diagnostics, Uithoorn, The Netherlands).

### Adhesion assay

*Ex vivo* generated NK cells were purified by CD56 selection. 1×10^5^ cells were suspended in RPMI-1640, and seeded onto confluent LecTERT cells in 12 well plates. The plates were rotated for 30 min at room temperature on a belly dancer. After extensive washing, all cells were released by trypsin, stained with CD56-APC and the number of CD56^+^ cells as well as total cells determined as described under the section Flow Cytometry. The adhesion capacity was recorded as percent of CD56^+^ cells within the total cells determined. The value obtained for NK cells differentiated without IL-12 was arbitrarily set to 100%.

### Cytotoxicity and IFN-γ-release assay

Flow cytometry-based cytotoxicity assays were performed as described previously [15]–[17]. Briefly, after incubation of 5×10^4^ effector cells (*ex vivo* NK cells from day 28 of culture) and 5×10^4^ target cells for 4 h at 37°C, cells were harvested and the number of viable target cells determined by flow cytometry using the fluorescent cell staining dye CFSE (carboxyfluorescein succinimidyl ester). Target cell survival was calculated as follows: % survival = {[absolute no. viable CFSE^+^ target cells co-cultured with NK cells]/[absolute no. viable CFSE^+^ target cells cultured in medium]}×100. The percentage specific lysis was calculated as follows: % lysis = {100-[% survival]}.

IFN-γ production of NK cells during coculture of effector and target cells was determined in the corresponding supernatants using ELISA (Pierce Endogen, Rockford, IL, USA). Absorbance was measured at 450 nm with a Multiscan MCC/340 ELISA reader (Titertek, Huntsville, Alabama, USA).

### Antibody-dependent cellular cytotoxicity assay using rituximab

The ADCC activity against two human acute lymphoblastic leukemia cell lines 721.221 and Nalm-6 was measured in triplicates within a Europium-release killing-assay as described previously [22]. Target cells were labelled with EuDTPA (europium diethylenetriaminopentaacetate), subsequently washed and incubated with 10 µg/ml rituximab (kindly provided by the pharmacy of the General Hospital Vienna, Austria) for 1 h at room temperature. After extensive washing 2×10^3^ target cells were incubated for 4 h with purified NK effector cells at various E∶T ratios in RPMI-1640 without phenol red (PAA Laboratories, Pasching, Austria) supplemented with 10% FCS. Maximal EuDTPA release was determined by incubation with 1% Triton X-100. Values for specific release of EuDTPA were determined with Delfia Enhancement Solution (Perkin Elmer, Brunn am Gebirge, Austria) via time-resolved fluorescence. The specific cytotoxicity was calculated as percent specific EuDTPA release = (Mean sample – Mean spontaneous release)/(Mean maximal release - Mean spontaneous release)×100.

### RNA preparation and cDNA synthesis

At least 1×10^5^ cells were lysed with Trizol (Invitrogen, Carlsbad, CA) and stored at −80°C. Total RNA was extracted according to the protocol provided by Invitrogen and 1 µg RNA was used to synthesize oligo-dT-primed cDNA with H Minus M-MuLV Reverse Transcriptase, following the manufacturer's protocol (enzyme and all reagents from Fermentas, St.Leon-Rot, Germany).

### Realtime RT-PCR analysis

Specific mRNA levels were measured by realtime RT-PCR starting from 100 ng cDNA using SYBR Green PCR Master Mix and a 7300 Realtime PCR system (Applied Biosystems, LifeTech, Vienna, Austria). Values were normalized to β-actin mRNA as internal standard and analyzed with StepOne software v2.1 (Applied Biosystems). Oligonucleotide primers were designed using the Primer-BLAST software (http://www.ncbi.nlm.nih.gov/tools/primer-blast/). Forward/reverse primers used were:

Eomes: CTGGCTTCCGTGCCCACGTC/CATGCGCCTGCCCTGTTTCG

ETS1: CCCCGTCCCCTTCCCCCTGTT/TTCCATATCCGGGGAGGGGAAAAGC

E4BP4: TTCTTTCTCCTCGCCGGCCCG/CGCCTGCTCCTTTTTGACGGTCTG

Gata-3: CAATGCCTGTGGGCTCTACT/TAAACGAGCTGTTCTTGGGG

ID2: TCAGCACTTAAAAGATTCCGTG/GACAGCAAAGCACTGTGTGG

ID3: GGCCCCCACCTTCCCATCCA/GCCAGCACCTGCGTTCTGGAG

IKZF3: CCTCGGAGATGGTTCCAGTT/CTGGCGTTCTTCATGGTTGC

Tbet: CACGTCCACAAACATCCTGT/GATCATCACCAAGCAGGGAC

TOX: AACCGGTGGACTGGAATAAC/TGGAGAACTGCCTTGACTGT

### Statistics

Results from single experiments performed in triplicates are described as mean ± standard deviation of the mean (SD). Combined results from several individual experiments including umbilical cord blood units from different donors are shown as mean ± standard error of the mean (SEM). Statistical analysis was performed using Student's t-test. A p-value of <0.05 was considered as statistically significant.

## Results

### Evidence for induction of CD62L, CD16 and KIR expression on *ex vivo* differentiated NK cells by low doses of IL-12

Initially, we have aimed to analyze the impact of several additional cytokines such as IL-12 or IL-21 on our recently established and characterized *ex vivo* human NK cell differentiation method, which includes IL-15 and IL-2 [15]–[17]. From these studies, we concluded that IL-12 had the most desirable effects on the NK cell generation procedure and should be further investigated. Therefore we performed a systematic analysis of the effects of IL-12 when applied in combination with IL-15 at day 10 of expansion from hematopoietic stem cells to induce NK cell differentiation. This was compared to the protocol adding only IL-15 at day 10. In all cultures from day 14 onwards IL-2 was provided in addition to direct further differentiation, proliferation and maturation (Figure S1A).

**Figure 1:**
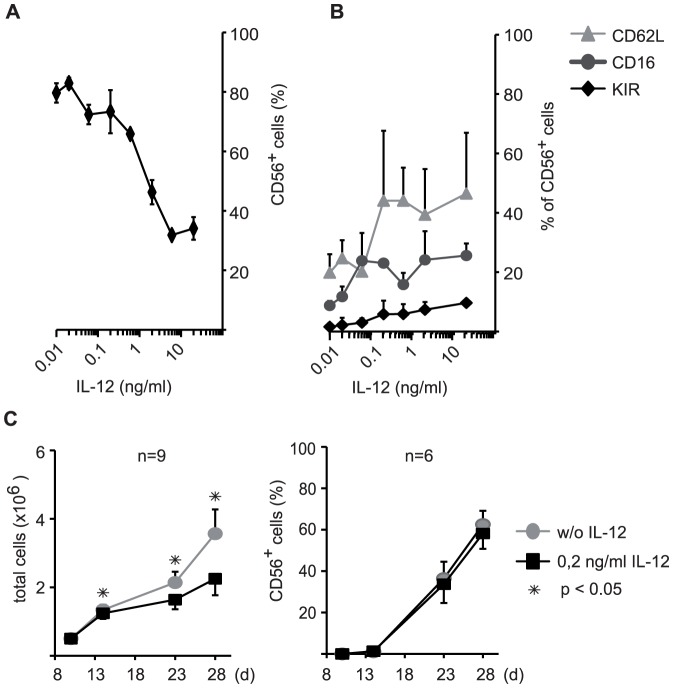
Effects of different concentrations of IL-12 on the phenotype and the purity of *ex vivo* generated NK cells. Effects of high and low doses of IL-12 on the *ex vivo* NK cell generation and the phenotype of the NK cells were determined by flow cytometry. In a dose-response analysis the effects of concentrations between 10 pg/ml and 20 ng/ml IL-12 on NK cell purity (A, displayed as % CD56^+^ NK cells in the culture) and NK cell receptor expression (B, displayed as % receptor positive cells within the CD56^+^ NK cell subset of the culture) were scored. Expression of CD62L, CD16 and KIR on CD56^+^ cells is depicted. Values are shown as mean ± SD calculated from triplicate wells for one representative experiment at day 28 of culture. (C) An optimal concentration of 0.2 ng/ml IL-12 was chosen for further experiments and analyzed at day 28 of culture. The increase in total cell number (left panel) and the purity of CD56^+^ NK cells (right panel), i.e. the percentage of CD56^+^ cells per total cell counts, was determined. Mean numbers of total cells or percentages of CD56^+^ cells ± SEM for several independent cultures (n) are shown as indicated.

To optimize the most useful concentration of IL-12 we first analyzed in detail the effect of IL-12 concentrations ranging from 10 pg/ml to 20 ng/ml on the NK cell differentiation procedure. Generally, increasing concentrations of IL-12 resulted in a reduced percentage of CD56^+^ NK cells in the cell culture at the end of the analyzed differentiation period at day 28 (Figure 1A). However, these CD56^+^ NK cells displayed CD62L, CD16 and KIR expression increasing with higher doses of IL-12 (Figure 1B). This dose-response analysis revealed that a concentration of 0.2 ng/ml IL-12 was sufficient to significantly enhance surface receptor expression on the *ex vivo* generated NK cells, but did not yet result in a significant reduction of the percentage of CD56^+^ cells in the generated cells at day 28 of culture. Moreover, in a time-dependent analysis of several independent cultures the defined optimal concentration of 0.2 ng/ml IL-12 revealed only a small reduction in total cell counts, but had no significant impact on the NK cell purity (% CD56 cells) achieved over the culture period (Figure 1C).

We have further tested different time points of addition of IL-12. However, when IL-12 was added later than day 10, e.g. at day 20, the effects on receptor expression on NK cells was not anymore detectable (Figure S1B), suggesting that IL-12 acts at an earlier stage of differentiation. Therefore, all further experiments were performed using 0.2 ng/ml IL-12 from day 10 onwards in addition to the other cytokines of the previously established NK cell generation system.

### Low dose IL-12 functions to increase mature phenotypic characteristics of *ex vivo* differentiated NK cells

Next we analyzed in more detail the impact of low dose IL-12 on the phenotype of the *ex vivo* differentiated NK cells in multiple experiments starting from different donors. In regard of the potential therapeutic use of the *ex vivo* generated NK cells we focused on receptors that are relevant for migratory capabilities of NK cells, e.g. important for NK cell homing into tissues, and on receptors that are connected to the inhibition or activation of the cytotoxic activity of NK cells and thus control NK activities.

**Figure 2:**
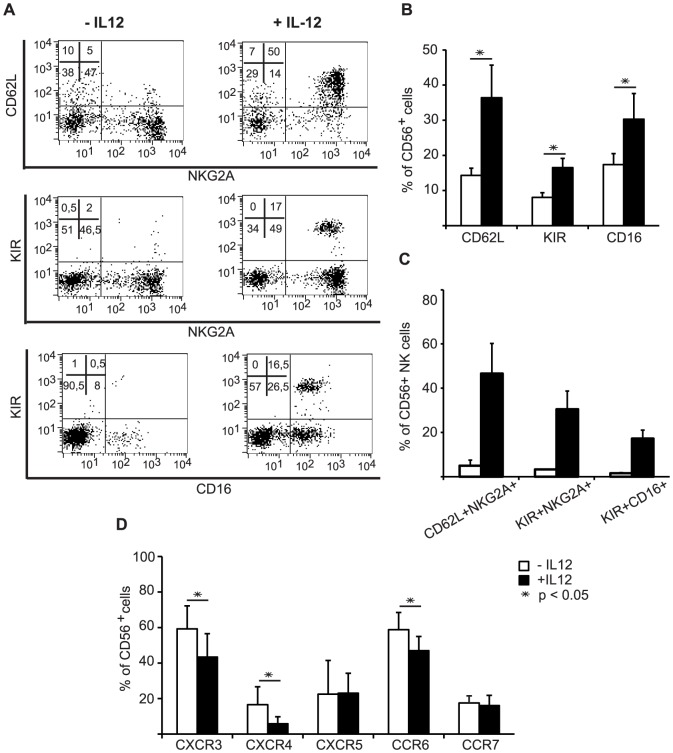
Influence of the optimized IL-12 concentration on receptor expression of *ex vivo* differentiated NK cells. The effect of 0.2/ml IL-12 on the expression of several NK cell antigens was determined by flow cytometry at day 28 of *ex vivo* differentiation. (A) Flow cytometry dot plots depicting the expression of CD62L, NKG2A, KIR and CD16 on gated CD56^+^ cells are shown for one representative *ex vivo* NK cell differentiation culture induced with or cultured without IL-12. (B) Column charts show for several independently performed cultures the IL-12-mediated increase of CD62L, KIR and CD16 positive cells and (C) of CD62L+NKG2A+, KIR+NKG2A+ and KIR+CD16+ double-positive cells. (D) The change of cell numbers with expression of the chemokine receptors CXCR3, CXCR4, CXCR5, CCR6 and CCR7 is shown. The statistical analyses are based on ≥5 independently performed experiments and are displayed as mean percentage ± SEM.

In this regard, the IL-12-containing protocol indeed led to a duplication of cells with expression of CD62L (L-Selectin) and of KIRs when measured in several independent experiments (Figure 2A and B). The pan-KIR antibody used to detect KIR expression binds to KIR2DL2, KIR2DL3, KIR2DS2 and KIR2DS4 and therefore is able to detect part of inhibitory and activating KIRs, which should be indicative of general KIR expression. Importantly, IL-12 addition also increased by more than 50% the number of cells with expression of FcRγIII/CD16, which is mediating antibody-dependent cellular cytotoxicity (Figure 2A and B). When we further analyzed cells double positive for CD62L and NKG2A or KIR and NKG2A or KIR and CD16 we noticed that IL-12 led to an over 10-fold increase in these double positive cells further substantiating the positive impact of IL-12 (Figure 2A and C).

We have also monitored the expression of lectin-like NK receptors in response to IL-12. The inhibitory CD94/NKG2A and the activating CD94/NKG2C were tested, but no significant influence of IL-12 on the prevalence of the CD94, NKG2A and NKG2C receptor chains was apparent within the time frame analyzed (Figure S2A). NKG2C displayed a donor-dependent variation, frequently nearly no expression to very few percent at day 28 of culture.

However, when we analyzed the chemokine receptor repertoire on *ex vivo* generated NK cells (Figure 2D), we noticed another significant effect of IL-12. Cells with surface expression of CXCR3, CXCR4 and CCR6 were diminished. No changes were observed for CXCR5 and CCR7, which were expressed at the low level of 20% of cells under both differentiation conditions.

When analyzed for individual experiments using different donors, the addition of IL-12 was, in the vast majority of cases, strongly increasing the number of CD62L, KIR and CD16 expressing NK cells, whereas it was reducing CXCR3, CXCR4 and CCR6 expressing NK cells (Figure 2D).

In summary, the data support that addition of IL-12 constitutes a significant improvement of the *ex vivo* differentiation system as it increases the percentage of *ex vivo* generated NK cells with receptor expression more similar to terminally differentiated peripheral blood NK cells.

### Cells with KIR and L-selectin expression reside within a CD33^+^/NKG2A^+^ population

**Figure 3:**
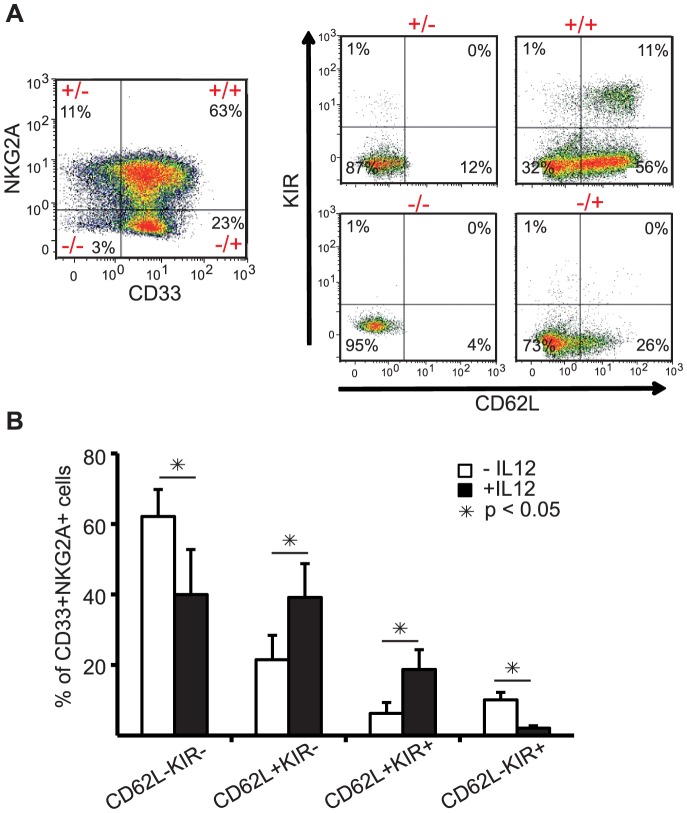
Effect of IL12 on CD62L and KIR expression within the developmental subset of CD33^+^NKG2A^+^ cells. *Ex vivo* NK cells differentiated with or without 0.2 ng/ml IL-12 were analyzed in regard of CD62L and KIR expression within the cell population displaying CD33 and NKG2A expression at day 28 of culture. (A) Flow cytometry dot plots of cells from an IL-12 containing culture are shown to illustrate the gating strategy. By gating on the four quadrants of the dot plot displaying NKG2A and CD33 expressing cells (left panel) the expression of KIR and CD56L on NKG2A^−^/CD33^−^, NKG2A^−^/CD33^+^, NKG2A^+^/CD33^+^ and NKG2A^+^/CD33^−^ cells is depicted (right panel). (B) displays the statistical analyses of the KIR and CD62L expressing cells within the NKG2A^+^CD33^+^ double positive cell population based on 5 independently performed cultures. Results are displayed as mean percentage ± SEM.

We recently described that developmental subsets of NK cells can be identified by the expression of CD33 and NKG2A [23]. In this model CD33 expression on NK progenitors precedes NKG2A expression. When we now analyzed the influence of IL-12 on the composition of the developmental stages characterized by the expression of CD33 and NKG2A we observed that the increased numbers of cells with expression of KIR and CD62L reside within the population of CD33^+^NKG2A^+^ NK cells (Figure 3A and B). This emphasizes that IL-12-mediated increased KIR and CD62L expression is largely initiated in the CD33^+^NKG2A^+^ developmental subset. However, no statistically significant differences were observed for the total number of cells within the subpopulations defined by CD33 and NKG2A expression in cultures with or without IL-12 (Figure S2B).

**Figure 4:**
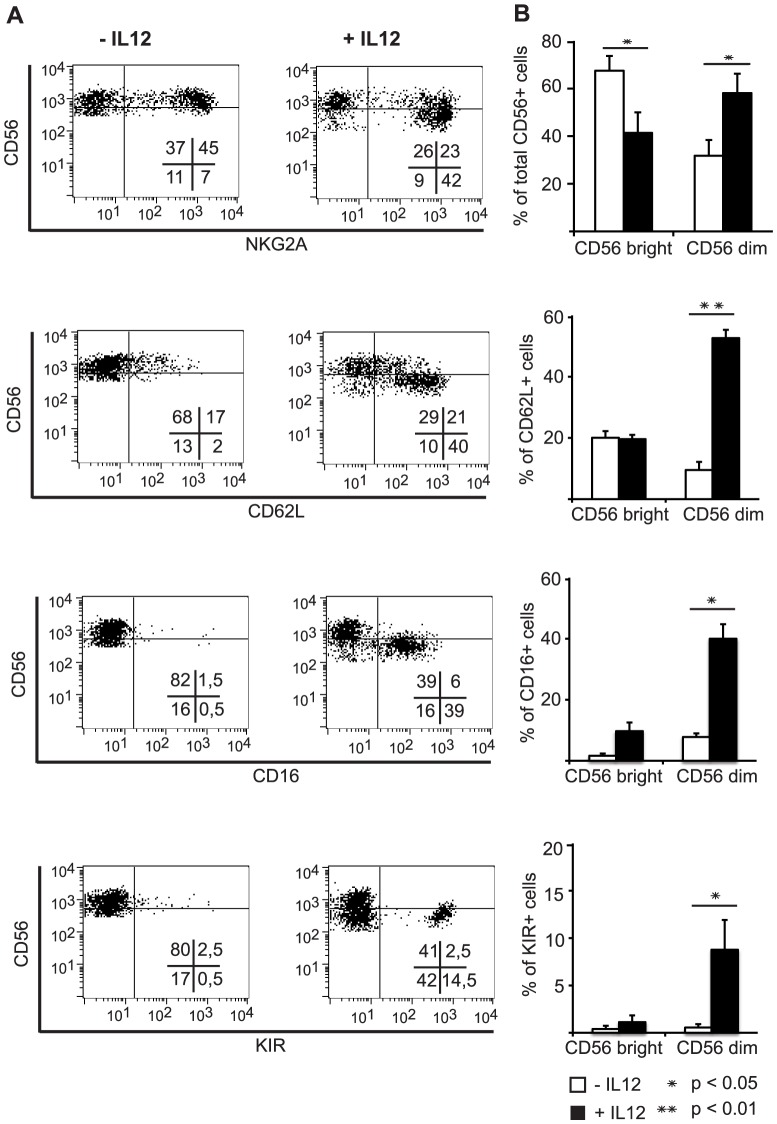
CD56 expression levels on IL-12-modulated *ex vivo* NK cells. The intensities of expression of CD56 on cells expressing CD62L, CD16 or KIR was analyzed by flow cytometry analysis on gated CD56^+^ *ex vivo* NK cells differentiated in presence or without IL-12. (A) Dot plots of one representative experiment of four that revealed strong induction levels for CD62L, KIR and CD16 is shown. (B) The statistical analyses of the CD56^bright^ and CD56^dim^ distribution for these four independent experiments is given from top to bottom for total CD56^+^, CD62L^+^NKG2A^+^, CD16^+^NKG2A^+^ or KIR^+^NKG2A^+^ cells. Results are displayed as mean percentage ± SEM.

In certain IL-12 modulated NK cell differentiation cultures, which exhibited particular high induction levels of CD62L, CD16 and KIR, we could further identify a reduction in the intensity of CD56 expression, although the CD56 expression levels of the *ex vivo* CD56^bright^ and CD56^dim^ cells were higher than typically observed for peripheral blood CD56^bright^ and CD56^dim^ NK cells. Flow cytometry analysis revealed, that in these cultures especially the cells with strong CD62L, KIR and CD16 expression displayed reduced CD56 intensity (Figure 4A and B).

All together these data indicate that IL-12 induces more advanced NK cell differentiation and maturation accompanied by reduced CD56 expression levels.

### IL-12 leads to significant changes in expression levels for Gata3, TOX and ID3 in *ex vivo* generated NK cells

**Figure 5:**
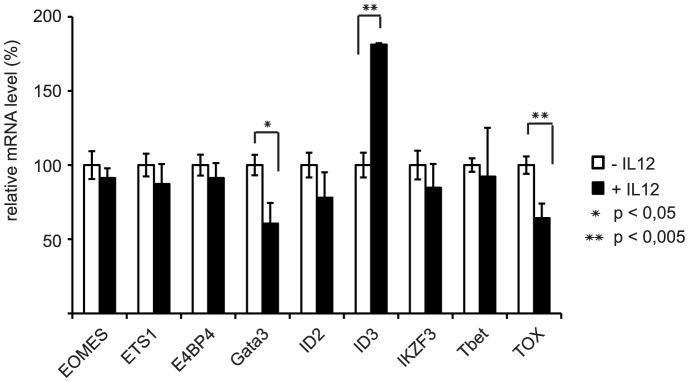
Regulation of transcription factors important for NK cell differentiation. Cultures of day 30 were MACS-sorted for CD56 expression. Total RNA was extracted and mRNA levels were analyzed by realtime RT-PCR for nine transcription factors implicated in NK cell differentiation. All values were normalized to β-actin as internal standard. Results are shown as mean values ± SEM calculated from 4 independent experiments performed. To display the results the mean values for the samples differentiated without IL-12 were arbitrarily set to 100%.

To gain insights into a potential molecular basis for the IL-12 effects on *ex vivo* differentiation of NK cells we performed a comparative real-time RT-PCR analysis to determine mRNA levels of several transcription factors in the *ex vivo* NK cell cultures developed in the presence or absence of IL-12. A series of nine transcription factors was chosen based on their strong upregulation in the *ex vivo* NK cell differentiation cultures as determined by gene profiling (D. Lehmann et al., in preparation) and on previous reports on their involvement in NK cell development [24]–[34]. The tested factors included E4BP4, Eomes, ETS1, Gata3, ID2, ID3, IKZF3, TOX and Tbet. From these nine transcription factors, three factors displayed significant changes, whereby Gata3 and TOX revealed significantly reduced mRNA expression levels, but ID3 mRNA level was significantly upregulated in the IL-12 cultures (Figure 5). This suggests that these factors could be involved in the observed phenotypic effects triggered by IL-12.

### *Ex vivo* NK cells generated with IL-12 display improved functional capacities

**Figure 6:**
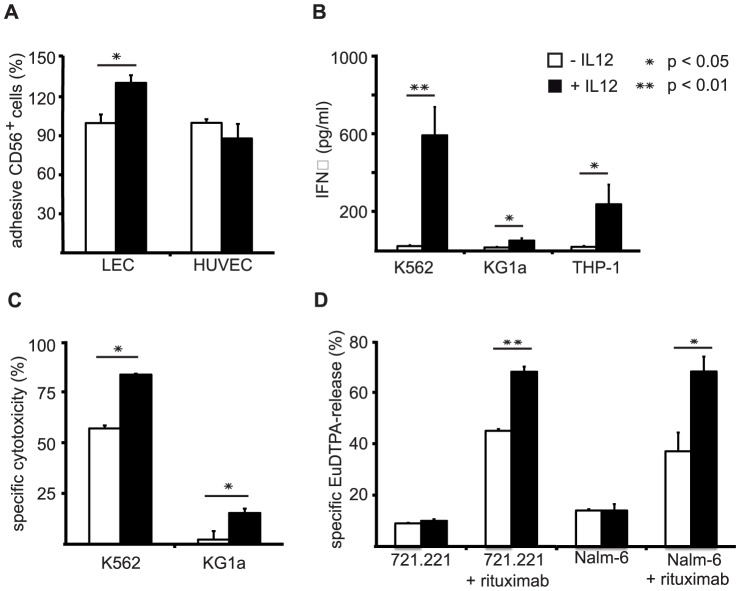
Functional assays display a correlation of phenotypical characteristics with improved functions regarding adhesion, cytokine production and cytotoxicity of IL-12-modulated NK cells. (A) Comparison of adhesion capacity to endothelial cells. *Ex vivo* generated NK cells from day 28 of cultures including or without IL-12 were purified and subsequently used in adhesion assays on LecTERT lymphatic endothelial cells (LEC) or resting human umbilical vein endothelial cells (HUVEC). Mean values ± SEM calculated from 3 independent experiments each performed in duplicates are shown. (B) Comparison of IFN-γ production capacities. *Ex vivo* generated NK cells from day 28 were cocultured with target cells at 1∶1 ratio. IFN-γ content was measured in the supernatants by ELISA. Mean values ± SEM calculated from 4 independent experiments are shown. (C) Comparison of NK cytotoxicity. After day 28 of culture *ex vivo* NK cells differentiated in the presence or absence of IL-12 were tested for their capacity to lyse the MHC class I-deficient target cell line K562 (n = 5) and the MHC class I-positive target cell line KG1a (n = 3). Cytotoxicity was determined on the basis of remaining intact CFSE-labeled target cells and is shown as mean values ± SEM. (D) Comparison of ADCC capacity. *Ex vivo* NK cells generated with or without 0.2 ng/ml IL-12 resulting in 36% or 21% CD56^+^CD16^+^ cells, respectively, were purified at day 28 of culture and subsequently analyzed in Europium-release killing assays. The human acute lymphoblastic leukemia cell lines 721.221 and Nalm-6 were used at an effector to target ratio of 6∶1 in the presence or absence of rituximab as indicated. Mean values ± SD calculated from triplicate wells are shown for one experiment representative of two performed.

We further were interested to examine whether NK cells generated in the presence of IL-12 would display better adhesion to lymphatic endothelial cells, since these express several ligands for CD62L. When we compared adhesion to lymphatic endothelial cells (LEC) and human umbilical vein endothelial cells (HUVEC) the data showed that *ex vivo* NK cells differentiated in the presence of IL-12 significantly better adhered to LEC than cells differentiated without IL-12, whereas no difference in adherence of these cells to unstimulated HUVEC was detectable (Figure 6A).

Furthermore, the effect of IL-12 on the IFN-γ producing capacity of the *ex vivo* NK cells was evaluated, because an enhancing effect of IL-12 on cytokine production by peripheral blood NK cells has been described. Upon coculture with the target cell lines K562, KG1a and THP-1 the release of IFN-γ by *ex vivo* NK cells was tested and an enhanced IFN-γ production was observed for IL-12-modulated *ex vivo* NK cells cocultured with all three target cell lines (Figure 6B).

Finally, we aimed to analyze if cells generated with IL-12 will display enhanced cytotoxicity. We tested the killing efficiency of the *ex vivo* NK cells using the MHC class I-negative, classical target cell line K562 and the MHC class I-positive cell line KG1a in a flow-cytometry based cytotoxicity assay. This analysis indeed revealed better natural cytotoxic capacities of the IL12-modulated *ex vivo* generated NK cells (Figure 6C).

The enhanced CD16 expression of the NK cells differentiated in the presence of IL-12 further suggested an increased potential for ADCC. Given the recent availability of therapeutic antibodies against different human malignancies synergistic effects of these antibodies with the IL-12-modulated *ex vivo* generated NK cells, e.g. after NK cell infusion, could be envisaged. Hence, we compared the killing efficiency of *ex vivo* NK cells generated with or without IL-12 for two B-cell leukemia-lines pretreated with the therapeutic B-cell-specific antibody rituximab. Both B-cell leukemia lines tested in a Europium-release killing assay, namely 7221.221 and Nalm-6, were significantly better lysed by NK cells when they were pretreated with rituximab. Moreover, IL12-modulated NK cells exhibited better killing capacities against both rituximab coated B-cell leukemia lines than cells generated without IL-12 (Figure 6D). The improved killing was in line with the observed increase from 21 to 36% CD16^+^ cells in the used batches of NK cells obtained without and with IL-12, respectively.

Thus, IL-12-generated NK cells reveal significant potential for therapeutic applications as they exhibit improved NK cytotoxicity as well as cytokine production and could be used in combination with available clinically approved antibodies.

## Discussion

The recently established *ex vivo* differentiation system developed by Glycostem Therapeutics for large scale generation of human NK cells is of interest for studies of human *ex vivo* NK cell development and holds great potential for adoptive immunotherapies of cancer [15]–[17]. Moreover, this system is easily amenable to modifications, which opens the possibility to study specific effects of individual cytokines on human NK cell differentiation and to generate tailored NK cell products with specified phenotypes and functions to specifically target malignancies.

We have now analyzed several cytokines for their impact on the *ex vivo* NK cell differentiation and found IL-12 to be an especially strong and useful modulator of this process. Importantly, under the influence of IL-12 NK cells acquired apparently more mature phenotypic and functional characteristics. This is indicated by the finding that the addition of IL-12 to the culture system at day 10 in combination with IL-15 caused a strong increase in cells with expression of KIRs and CD16, i.e. receptors upregulated late in maturation, and a down-modulation of cells displaying CXCR3, CXCR4 and CCR6, which have been described to be lower expressed on mature NK cells [1]–[3], [6]–[8] (Figure 2). As reported by us previously [15]–[17], at day 10 of culture about 20% of cells are CD14^+^ monocytic cells, which expand further until day 15 before reaching a plateau phase and declining at the end of culture. In contrast, CD56^+^ NK cells can be detected beginning from day 17 and further accumulate to represent 60–70% of cells at day 30 and over 95% after 6 weeks of culture. It is therefore possible that IL-12 exerts its effect directly on NK cells and their progenitors or indirectly via stimulating CD14 cells. In this regard, realtime RT-PCR analysis of mRNA expression for IL12RB2, the signaling chain of the dimeric IL-12 receptor, revealed significant IL-12RB2 mRNA levels only within CD56^+^ cells but not within the CD14^+^ monocytic cells or CD56^−^CD14^−^ cells present in the culture (Figure S3A). Combined with our observation that IL-12 added at day 20 of culture would not lead to comparable effects (Figure S1B), this suggests that IL-12 may act primarily on NK cell progenitors starting to express CD56 and within the first days thereafter.

Similar to studies of IL-12 induction on peripheral blood NK cells we observed significant cell death within the *ex vivo* differentiation cultures in correlation to increasing IL-12 concentrations [35]. It was therefore important that we defined an optimized low IL-12 concentration that leads to an improved NK cell phenotype while at the same time maintaining proliferation and survival of cells and ensuring the final purity of the NK cells (Figure 1).

Previous studies have revealed that IL-12 induced peripheral blood NK cells can acquire more cells with CD56^bright^ expression as well as exhibit a mature differentiated NK cell phenotype depending on the specific conditions employed. On the one hand it was described that IL-12 induced a CD56^bright^ NK cell phenotype including up-regulation of CD94 and CD62L and a down-modulation of CD16 [11]. On the other hand, it was reported that CD16^−^CD56^+^ PBNK cells treated with IL-12 in combination with IL-2 and IL-15 developed more CD16 expression alongside with CD56^bright^ expression [36]. Both studies described the impact of high concentrations of IL-12 on NK cell receptor expression and function in mature peripheral blood NK cells. When we tested induction of peripheral blood NK cells with a concentration of 0.2 ng/ml IL-12 for 6 days in differentiation medium only a significant upregulation of CD62L, but no changes in expression patterns for KIR and CD16 were revealed (data not shown) supporting that low dose IL-12 has to be present during differentiation of NK cells to upregulate these receptors.

Moreover, others have dissected human NK cell subsets on the basis of CD56 and CD16 expression and suggested that CD56^bright^CD16^+^ NK cells represent an intermediate stage of NK cell maturation between CD56^bright^CD16^−^ and CD56^dim^CD16^+^ NK cells already exhibiting mature functional capacity [37]. These data are in coherence with the findings, that in our system IL-12 leads to an increased expression of CD16 and KIRs (Figure 3) on NK cells that, as we described previously [15], highly express CD56. Furthermore, these cells are highly functional in terms of cytokine production and cytotoxicity (Figure 6B–D).

Recently, within a different study, we have identified distinct stages of human NK cell development on the basis of CD33 and NKG2A expression [23]. We defined a subset of CD33^+^NKG2A^+^ NK cells to constitute a developmentally more mature NK cell population than CD33^+^NKG2A^−^ NK cells. When we here tested the prevalence of CD62L and KIR expressing NK cells in the CD33^+^NKG2A^+^ NK cell population, the IL-12-mediated increase of CD62L^+^ and CD62L^+^KIR^+^ NK cells was confined to this subset (Figure 3). This strongly supports that the further maturation induced by IL-12 occurs in the CD33^+^NKGA^+^ population. In addition, we observed that the IL-12-induced process can be accompanied by a reduction in CD56 levels indicating that these cells may develop towards CD56^dim^ NK cells (Figure 4).

Specific chemokine receptors guide NK cells into lymphoid and non-lymphoid tissues and sites of tissue inflammation along chemotactic gradients, therefore the pattern of chemokine receptor expression can be correlated with specific NK cell subtypes [2], [6]–[8]. For example CCR7, that guides CD56^bright^ NK cells into secondary lymphoid organs, was expressed at low levels on *ex vivo* differentiated NK cells independent of the influence of IL-12. Furthermore, *ex vivo* NK cells differentiated in the presence of IL-12 express reduced levels of the chemokine receptor CXCR3 and CCR6 (Figure 3). Taking into consideration that others have described that CXCR3 and CCR6 are typically higher expressed by CD56^bright^ peripheral blood NK cells and lower by CD56^dim^ peripheral blood NK cells, the observation of decreased expression of CXCR3 and CCR6 by IL-12 modulated NK cells is in line with further maturation.

Furthermore, whereas IL-12-induced KIR and CD16, but -lowered CXCR3 and CCR6 expression, as well as the reduced CD56 levels clearly favor the idea of more mature NK cells, the induction of CD62L by IL-12 during *ex vivo* NK cell differentiation can not immediately be connected to maturation (Figure 2 and 3). CD62L is usually high on CD56^bright^ cells [2]. However, a recent study revealed that CD62L^+^CD56^dim^ PBNK cells exhibit the full functional repertoire of NK cell cytokine production and cytotoxicity and are likely also representing an intermediate stage of NK cell differentiation towards fully cytotoxic CD56^dim^CD62L^−^ NK cells [38]. CD62L is an important receptor guiding NK cells in and out of lymph nodes through interactions with ligands on high endothelial venules and, e.g. by binding to the ligand mannose receptor (MMR), along afferent and efferent lymphatic endothelium [39], [40]. Furthermore, CD62L also mediates rolling of leukocytes on activated endothelium for extravasation into inflamed tissue [41], [42]. In agreement with the known functions of CD62L we could show, that high expression of CD62L on IL-12 differentiated NK cells is correlated with better adhesion to lymph endothelial cells but not to resting vascular endothelial cells (HUVECs) (Figure 6A).

Early studies already indicated the potency of IL-12 to modulate NK differentiation towards IFN-γ producing and cytotoxic NK cell [43]. In recent years, additional evidence in patients with dysfunctions in IL-12-signaling pathways was obtained revealing the necessity of NK cell priming through IL-12 for the acquisition of functional activity [44]. The acquisition of IFN-γ producing and cytotoxic NK cell functions following IL-12 treatment was correlated with induced expression of the IFN regulating factor-1 (IRF-1) and perforin genes [45], [46]. Our results show clearly increased IFN-γ production for IL-12-differentiated NK cells (Figure 6B) and in realtime RT-PCR analyses we could confirm an upregulation of perforin mRNA levels alongside with increased CD62L and CD16 mRNA levels upon addition of IL-12 to the *ex vivo* cultures (Figure S3B). In line with this, our *in vitro* killing assays using the MHC class I-positive KG1a and the MHC class I-negative K562 cell lines confirmed enhanced cytotoxicity of *ex vivo* NK cells differentiated with IL-12 and supports their improved potential for antitumor therapies (Figure 6C).

A characteristic of CD56^dim^ NK cells is the capability to lyse antibody-coated target cells, a phenomenon known as ADCC which is mediated through the receptor CD16/FcRγIII. The enhanced expression of CD16 generated under the influence of IL-12 might therefore be utilized in therapeutic settings combining the cytotoxic activity of *ex vivo* NK cells with antibodies against malignant cells. Previous studies revealed the potential and importance of the clinically approved antibody rituximab recognizing CD20 on B-cell leukemias in combination with human peripheral blood NK cells [18], [47]. In this regard we could confirm increased ADCC activity of the IL-12 modulated *ex vivo* NK cells against two B-cell lines coated with rituximab (Figure 6D). This substantiates the improved functional capacity and potential therapeutic utilization of these cells in combination with therapeutic antibodies.

IL-12 presumably affects differentiation of NK cells via regulation of specific transcription factors. It has been shown that within the lineage specification towards NK cell development the transcription factors E4BP4 (NFIL3), Ets-1, ID2 and ID3 possess great impact as master switches [24], [26]–[29]. Furthermore, TOX and IKZF3 (AIOLOS) are crucial for T and NK cell development [31], [34]. Moreover, homing and cytokine production capacities as well as the expression of the inhibitory NKG2A receptor depend on the zinc finger transcription factor GATA-3 [30], [32]. Terminal maturation and homeostasis of NK cells is controlled by T-bet and the related factor EOMES [33]. When we tested these nine transcription factors implicated in NK cell development, we observed that cultures with addition of IL-12 displayed increased expression of ID3, whereas Gata3 and TOX were downregulated. All others showed no significant changes in expression level (Figure 5). This suggests that ID3, Gata3 and TOX might be involved in mediating IL-12 induced phenotypic and functional characteristics of human *ex vivo* NK cells.

Altogether, our findings indicate that IL-12 can modulate NK cell differentiation and is a promising additive for human *ex vivo* NK cell differentiation that can be exploited to generate NK cells with improved properties. This may hold significant potential for the use of these cells in the treatment of leukemias as straightforward therapeutics alone, or in combination with antibodies aiming at ADDC, and could include conditioning regimens pre- or post stem cell transplantation. Similarly, outside the field of hematology, these cells hold promise for late stage interventions in solid tumors.

## Supporting Information

Figure S1 **Scheme of** ***ex vivo*** **NK cell differentiation protocol and effects of addition of IL-12 at different time points.** (A) The principle of (a) the previously established [16], [17] as well as (b) the IL-12-modulated protocol for *ex vivo* hematopoietic stem cell (HSC) expansion and NK cell differentiation is indicated. In the basic protocol CD34^+^ UCB cells are expanded using SCF, IL-7, TPO, Flt3L, G-CSF, GM-CSF, IL-6 and low molecular weight heparin in the culture medium for 10 days. This is followed in the standard protocol by replacement of TPO with IL-15 at day 10 to initiate NK cell differentiation. Furthermore, at day 14 Flt3L is replaced by IL-2 in medium without the low molecular weight heparin. In the modified protocol a low concentration of IL-12 is given together with IL-15 from day 10 on, otherwise the procedure is identical. Cells were cultured for 28 days. (B) The effect of IL-12 addition at day 10 or day 20 on the number of NK cells with CD62L, CD16 or KIR expression analyzed at day 28 is shown. Flow cytometry was performed using triplicate wells. One representative experiment of three performed is shown. Data are displayed as mean ± SD.
(EPS)

Figure S2 **Effects of IL-12 on lectin-like receptors and CD33^+^ subsets.** (A) Effects of IL-12 on expression of the lectin-like NK receptors CD94/NKG2A and CD94/NKG2C. The effect of 0.2 ng/ml IL12 on the expression of the CD94, NKG2A and NKG2C receptor chains was analyzed by flow cytometry on gated CD56^+^ cells. (B) Influence of IL-12 on total cell numbers for the different developmental subsets defined by CD33 and NKG2A expression. *Ex vivo* NK cells differentiated with or without 0.2 ng/ml IL-12 were analyzed in regard of CD33 and NKG2A expression by flow cytometry on gated CD56^+^ cells. The statistical analyses are based on 4 independently performed experiments and are displayed as mean percentage ± SEM.
(EPS)

Figure S3 **Expression of IL-12 receptor and perforin mRNAs by** ***ex vivo*** **NK cells.** (A) IL-12 receptor mRNA. Cultures were sorted into CD14^−^CD56^−^, CD14^+^, CD56^+^NKG2A^−^ and CD56^+^NKG2A^+^ cells as previously described (15). Total RNA was extracted and mRNA levels were analyzed by realtime RT-PCR for IL12 receptor mRNA. All values were normalized to β-actin as internal standard. Results are shown as mean values ± SD calculated from 1 representative experiment of two performed. (B) Perforin mRNA. Cultures differentiated in the presence or absence of IL-12 were used for RNA extraction at day 28 followed by realtime RT-PCR analysis. Results are shown as mean values ± SEM calculated from 3 independent experiments.
(EPS)
